# Bacterial Carriage of Genes Encoding Fibronectin-Binding Proteins Is Associated with Long-Term Persistence of Staphylococcus aureus in the Nasal and Gut Microbiota of Infants

**DOI:** 10.1128/AEM.00671-21

**Published:** 2021-07-13

**Authors:** Forough L. Nowrouzian, Annika Ljung, Bill Hesselmar, Staffan Nilsson, Ingegerd Adlerberth, Agnes E. Wold

**Affiliations:** a Institute of Biomedicine, Department of Infectious Diseases, The Sahlgrenska Academy, University of Gothenburg, Gothenburg, Sweden; b Institute of Clinical Science, Department of Paediatrics, The Sahlgrenska Academy, University of Gothenburg, Gothenburg, Sweden; c Institute of Biomedicine, Department of Laboratory Medicine, The Sahlgrenska Academy, University of Gothenburg, Gothenburg, Sweden; The Pennsylvania State University

**Keywords:** infants, *S. aureus*, adhesins, enterotoxin, fibronectin-binding protein, gut colonization, nasal colonization, persistence, virulence factors

## Abstract

Staphylococcus aureus can colonize both the anterior nares and the gastrointestinal tract. However, colonization at these sites in the same individuals has not been studied, and the traits that facilitate colonization and persistence at these sites have not been compared. Samples from the nostrils and feces collected on 9 occasions from 3 days to 3 years of age in 65 infants were cultured; 54 samples yielded S. aureus. The numbers of nasal and fecal S. aureus strains increased rapidly during the first weeks and were similar at 1 month of age (>40% of infants colonized). Thereafter, nasal carriage declined, while fecal carriage remained high during the first year of life. Individual strains were identified, and their colonization patterns were related to their carriage of genes encoding adhesins and superantigenic toxins. Strains retrieved from both the nose and gut (*n* = 44) of an infant were 4.5 times more likely to colonize long term (≥3 weeks at both sites) than strains found only in the rectum/feces (*n* = 56) or only in the nose (*n* = 32) (*P* ≤ 0.001). Gut colonization was significantly associated with carriage of the *fnbA* gene, and long-term colonization at either site was associated with carriage of *fnbA* and *fnbB.* In summary, gut colonization by S. aureus was more common than nasal carriage by S. aureus in the studied infants. Gut strains may provide a reservoir for invasive disease in vulnerable individuals. Fibronectin-binding adhesins and other virulence factors may facilitate commensal colonization and confer pathogenic potential.

**IMPORTANCE** S. aureus may cause severe infections and frequently colonizes the nose. Nasal carriage of S. aureus increases 3-fold the risk of invasive S. aureus infection. S. aureus is also commonly found in the gut microbiota of infants and young children. However, the relationships between the adhesins and other virulence factors of S. aureus strains and its abilities to colonize the nostrils and gut of infants are not well understood. Our study explores the simultaneous colonization by S. aureus of the nasal and intestinal tracts of newborn infants through 3 years of follow-up. We identify bacterial virulence traits that appear to facilitate persistent colonization of the nose and gut by S. aureus. This expands our current knowledge of the interplay between bacterial commensalism and pathogenicity. Moreover, it may contribute to the development of targeted strategies for combating S. aureus infection.

## INTRODUCTION

Staphylococcus aureus is one of the most important human pathogens, causing infections that range from minor skin conditions to life-threatening diseases such as septicemia (1). S. aureus is also a member of the commensal flora, colonizing the anterior nares of approximately 50% of the population, with 20% being persistently colonized (2, 3). Infection by S. aureus is generally preceded by commensal colonization (1, 4, 5); being a nasal carrier of S. aureus increases the risk of invasive infection 3-fold (6).

S. aureus may also colonize the mucosal surfaces of the throat, vagina, and gut (7, 8). During the last few decades, S. aureus has been identified as a common colonizer of the gut of infants (7, 9). It has been proposed that this is because improved levels of hygiene have decreased colonization by more “traditional” fecal bacteria (10). Thus, S. aureus is found in the feces of approximately 75% of Swedish infants, with individual strains often persisting in an infant for many months (9). In S. aureus-colonized infants, the bacterium attains high fecal population levels (10^7^ CFU/g, on average, in 1-month-old infants) (9). The gut has been highlighted as a potentially important reservoir for S. aureus strains that cause infections (7, 11, 12). Accordingly, carriage of S. aureus in both the nose and gut seems to increase the risk of developing S. aureus infection compared to nasal carriage alone (12).

Staphylococcal virulence factors enable the bacterium to cause disease (13). These factors may also enable commensal colonization and persistence by staphylococci (1, 4, 5). Staphylococcal adhesins enable the bacteria to adhere to host matrix components, such as collagen, fibronectin, elastin, laminin, and bone sialoprotein (14). Fibronectin-binding proteins (15–18) and collagen-binding protein (19) have been shown to contribute to virulence in experimental invasive disease models, and their respective genes (*fnb* and *cnb*) are frequently detected in S. aureus isolates from the nostrils, from skin infections, and from persons with invasive disease (20, 21). Nasal commensal S. aureus strains actively transcribe *fnbA* (22). For gut-colonizing S. aureus strains, carriage of the *cnb* gene is associated with increased numbers of S. aureus in the feces (23).

The plasma-clotting protein fibrinogen is recognized by several staphylococcal adhesins, i.e., clumping factors A and B (ClfA and ClfB) and fibrinogen-binding proteins A and B. ClfA has been shown to contribute to septicemia in a murine model (24). ClfB also mediates adherence to epidermal cytokeratins (25). S. aureus strains from the nostrils spontaneously transcribe *clfB* (22), and *clfB* facilitates experimental nasal colonization in humans (26).

Other virulence factors of S. aureus include various toxins. T-cell-mitogenic toxins or superantigens, e.g., S. aureus enterotoxin A (SEA), SEB, SEC, etc., and toxic shock syndrome toxin-1 (TSST-1) (27), have been shown to contribute to disease in various experimental models (28). The most commonly detected superantigens, SEG, SEI, SElM, SElN, and SElO, all of which are encoded by the *egc* gene cluster (29), appear to have lower pathogenic potential (28, 30). Both nasal commensals and skin infection isolates frequently carry genes that encode the superantigens SElM-SElO, SEG, and SEI (31). These toxins may play roles in commensal colonization, as S. aureus strains that possess the genes for the enterotoxins M/O (encoded by the *egc* cluster) attain higher fecal numbers than strains that lack these genes (23).

Compared to nasal colonization by S. aureus, less is known regarding its gut-colonizing behavior, and the nasal and gut colonization profiles have not been studied in the same individuals. Similarly, the factors that enable colonization of these two separate ecological niches and long-term persistence at these sites have not been elucidated.

Here, we examined the patterns of S. aureus nasal and fecal colonization from 3 days to 3 years of age in 54 culture-positive infants who harbored the species in the nose, gut, or both the nose and gut. Individual strains were identified, and their virulence gene patterns were related to the site of colonization and the capacity of the strain to persist at that anatomical site.

## RESULTS

### Comparison of S. aureus colonization rates in the nose and gut of infants.

**FIG 1:**
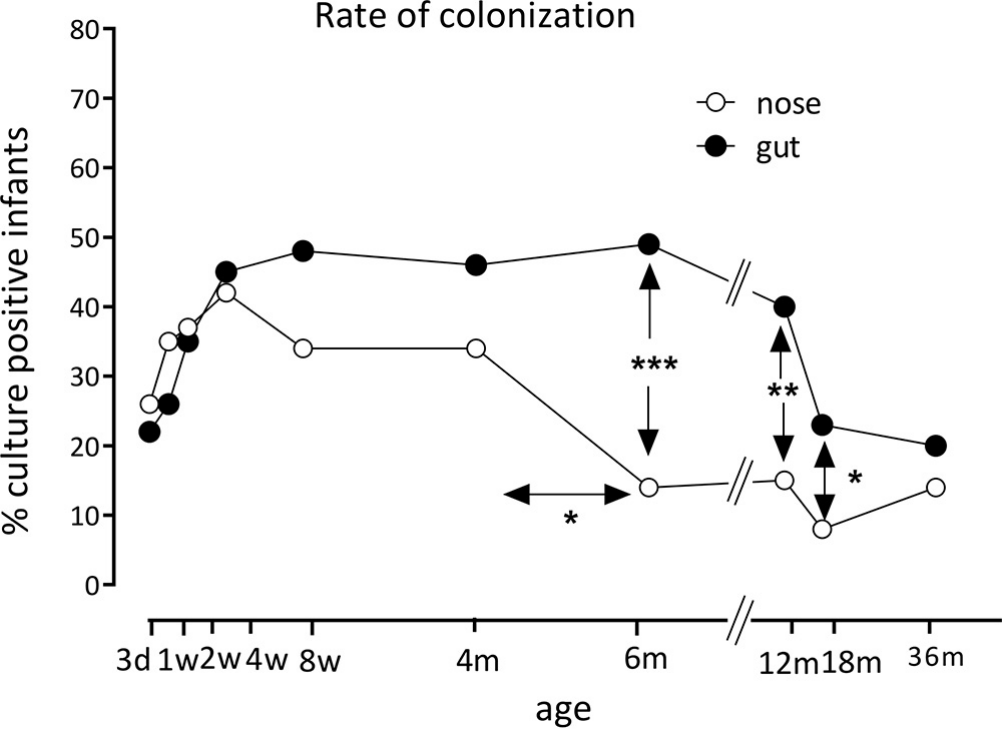
S. aureus colonization rates in the nasal and gut microbiota of 65 infants who were followed from 3 days of age to 3 years of age. Nasal colonization (open circles) was assessed by culturing nasal swabs. The *y* axis label is percent S. aureus culture-positive infants. The gut colonization rate (filled circles) was assessed from rectal swabs at 3 days of age and from fecal samples at subsequent time points. Asterisks refer to significant differences between the nasal and gut colonization rates at certain time points (indicated by vertical arrows) or significant differences in nasal colonization between two time points (indicated by horizontal arrows). Proportions were compared using Fisher’s exact test.

Figure 1 compares the rates of S. aureus colonization of the nose and gut in the 65 infants between samples taken at 3 days and 3 years of age. Nasal and gut colonization by S. aureus increased rapidly in parallel over the first weeks of life (Fig. 1). Thereafter, nasal colonization declined progressively, from a peak level at 1 month of age (42% of infants colonized) to 14% by 6 months of age and 8% by 18 months (4 months versus 6 months of age, 34% versus 14%, *P* = 0.01). In contrast, the fecal colonization rate remained high, at slightly below 50%, up to 1 year of age (Fig. 1). Thus, S. aureus was significantly more frequently retrieved from feces than from nasal swabs at 6, 12, and 18 months of age (*P* = 0.0001, *P* = 0.002, and *P* = 0.01, respectively) (Fig. 1).

In total, 54 infants (83%) yielded at least one sample that contained S. aureus. The absolute majority (71%, 46/65) had S. aureus in both the nose and rectum/feces, while four infants (6%) had S. aureus only in the nose and another four infants (6%) had S. aureus only in the rectum/feces. The proportion of infants colonized by S. aureus on at least one sampling occasion did not differ between infants from farming and nonfarming families, and colonization was not linked to the infant’s sex, delivery mode, or antibiotic treatment (Table 1). Breast-feeding was associated with reduced rate of S. aureus colonization in the infants (Table 1).

**TABLE 1:** Characteristics of the FARMFLORA cohort

Parameter	No. (%) of infants		*P* value^*d*^
	All infants (*N* = 65)	S. aureus-colonized infants (*N* = 54)	
Farmer’s children	28 (43)	23 (43)	1.0
Pets in household^*a*^	40 (62)	32 (60)	0.5
Vaginal delivery	55 (85)	44 (81)	0.2
Exclusive breastfeeding at 4 mo of age	34 (52)	25 (46)	0.04
Partial breastfeeding at 12 mo of age	33 (51)	24 (44)	0.01
First-born	29 (45)	27 (50)	0.09
Intrapartum antibiotics^*b*^	10 (15)	10 (18)	0.2
Antibiotics, 0–6 mo of age^*c*^	11 (17)	7 (14)	0.09
Antibiotics, >6–12 mo of age	11 (17)	10 (18)	0.6
Antibiotics, >12–18 mo of age	17 (26)	16 (30)	0.2
Girls	32 (49)	27 (50)	1

a Data regarding household pets (cat and/or dog) were missing in one case.

b Antibiotic treatment of the mother during partus. The administered antibiotics included erythromycin (number of infants [*N*] = 2), cefuroxime (*N* = 2), metronidazole (*N* = 2), benzylpenicillin (*N* = 1), and benzylpenicillin plus cefuroxime (*N* = 1). Missing data, *N* = 2.

c Antibiotic treatment of the child at least once in the indicated time period. The following antibiotics were administered: at 0 to 6 months of age, phenoxymethylpenicillin (*N* = 4), ampicillin (*N* = 1), cephalosporin (*N* = 1), and trimethoprim-sulfamethoxazole (*N* = 3); missing data, *N* = 2; at 6 to 12 months of age, phenoxymethylpenicillin (*N* = 10), ampicillin (*N* = 3), cephalosporin (*N* = 1), and isoxazolyl penicillin (*N* = 1); and at 12 to 18 months of age, phenoxymethylpenicillin (*N* = 13), trimethoprim (*N* = 1), trimethoprim-sulfamethoxazole (*N* = 4), ampicillin (*N* = 1), amoxicillin (*N* = 2), amoxicillin-clavulanic acid (*N* = 2), and isoxazolyl penicillin (*N* = 2).

d *P* values refer to the comparisons of infants who were colonized and those who were not colonized by S. aureus on at least one sampling occasion.

### Identification of S. aureus strains and characterization of their colonizing behaviors.

**FIG 2:**
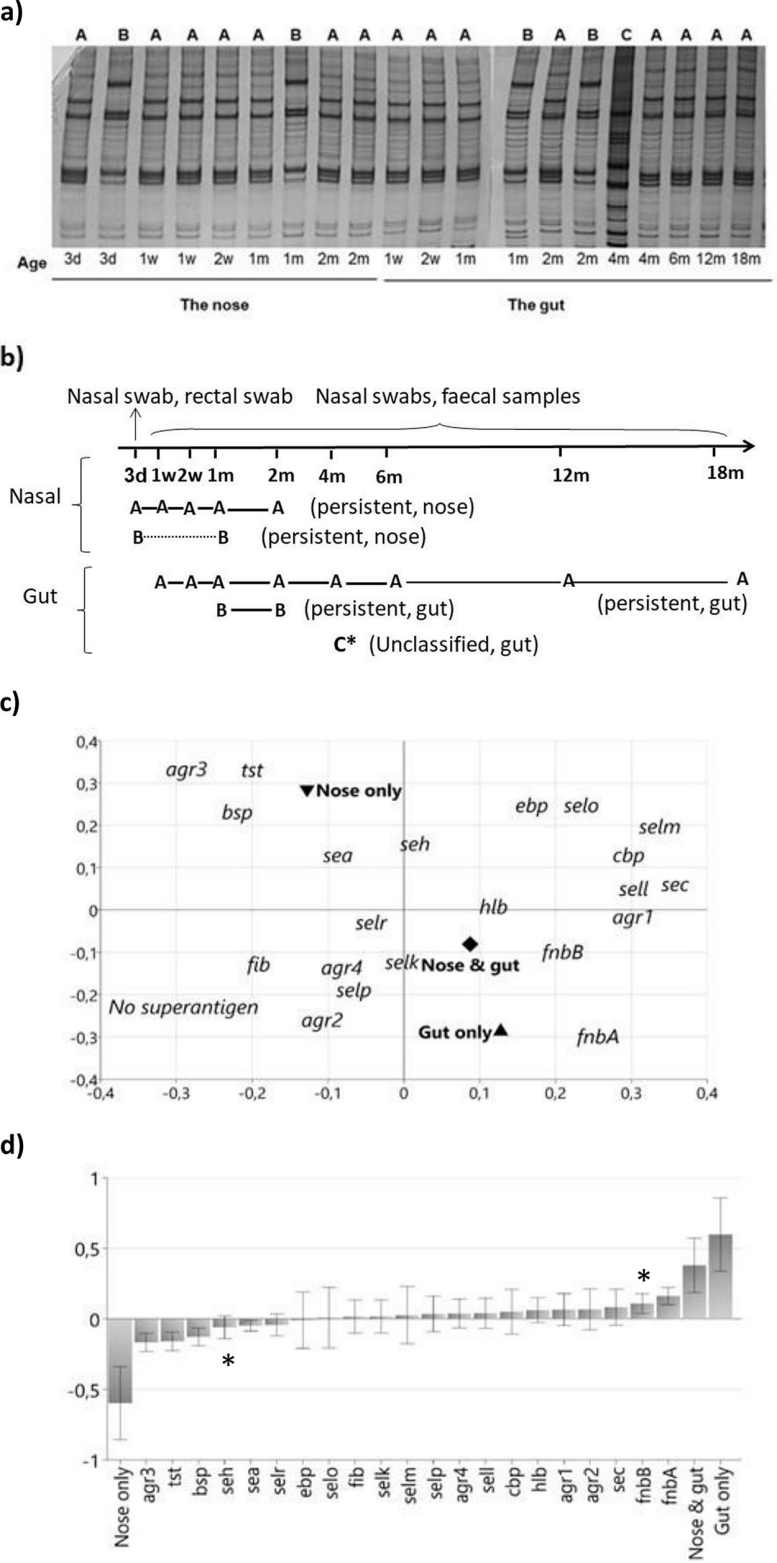
(a) RAPD patterns of 9 nasal and 11 gut S. aureus isolates obtained on different occasions from a single infant. These isolates were subjected to RAPD, and the patterns were visualized by silver staining. Isolates that show identical RAPD patterns are considered to belong to the same strain. Three individual strains were identified, termed A, B, and C. Strains A and B were repeatedly isolated from both the nose and gut, while strain C was only isolated once, from the gut. (b) Visualization of the colonization pattern of the child colonized by the strains described in panel a. Strain A was repeatedly found in cultures of nasal swabs acquired from 3 days to 2 months of age and in fecal cultures taken from 1 week to 18 months of age. Thus, strain A was persistent in both the nasal and gut microbiota. Strain B, which was also found in both the nasal (from 3 days to 1 month of age) and gut (from 1 to 2 months of age) microbiota, was also defined as a persistent strain at both sites. Strain C, which appeared only once in the fecal sample taken at 4 months of age, could not be characterized as either persistent or transient due to the long interval between sampling occasions. (c) PCA showing S. aureus virulence gene carriage in relation to site of colonization (nose only, gut only, or nose and gut). Virulence genes that encode toxins or adhesins and the four *agr* alleles (genetic background) are included in the analysis. Virulence genes found in <5% or ≥98% of the strains were excluded. The following toxin genes were identified in <5% of the strains: *sed* (enterotoxin D), *eta*, *etb*, and *etd* (exfoliative toxins A, B, and D), *pvl* (Panton-Valentine leukocidin), and *lukM* (leukotoxin M). No strain carried the genes for *seb* (enterotoxin B) or *selq* (enterotoxin Q). The following adhesin genes were carried by ≥98% of the strains: *clfA*, *clfB*, and *lbp* (laminin-binding protein). No superantigen denotes the absence of all screened superantigen genes. (d) OPLS-DA analysis showing the traits that are characteristic of strains that colonized only the gut as well as those found in both the nose and gut of an infant (positioned on the right side of the diagram) and traits that characterize strains found exclusively in the nose (listed on the left side). Virulence genes that encode toxins and adhesins and the four *agr* alleles (genetic background) were included in the analysis. The height of each bar shows the explanatory power of the variable, and the error bar indicates the uncertainty of its contribution. Virulence genes found in <5% of the strains or ≥98% of the strains were excluded (as described for panel c). Asterisks located above or below a variable bar indicate that this variable is significantly associated with the colonization site in a univariate analysis (Fisher’s exact test) after correction for multiple comparisons.

In all, 449 S. aureus isolates from the 54 colonized infants were analyzed by RAPD to identify distinct strains in individual infants. Figure 2a shows an example of the RAPD patterns of the 9 nasal and 11 gut isolates of an infant. Two strains, designated strains A and B, were found in the nose, and both appeared in the 3 day sample; strain A was present until 2 months of age, and strain B persisted until 1 month of age. Strain A was also found in the gut, where it was present from 1 week to 18 months of age. Strain B was likewise found in the gut (at 1 and 2 months of age). Furthermore, a third strain (strain C) appeared in a single fecal sample, obtained at 4 months of age (Fig. 2a). The colonization patterns of strains A, B, and C are depicted in Fig. 2b. Both strain A and strain B persisted in the nasal microbiota, i.e., were present for ≥3 weeks at the colonized site. Both strains were also persistent colonizers of the infant gut, where strain A colonized from 1 week to 18 months of age. No transient strain was identified in this infant, i.e., a strain that colonized for <3 weeks. Strain C was deemed “unclassified” with respect to persistence. As it was only present in one sample and this sample was obtained during a period of sampling with long intervals, it could not be classified as either persistent or transient.

Thus, each strain could be classified as persistent, transient, unclassified, or not present at each site, which yielded 15 possible combinations, 13 of which were represented among the 132 identified strains (see Table S1 in the supplemental material). Table 2 summarizes the colonization behaviors of the S. aureus strains: 24% (number of strains [*n*] = 32) were found only in the nose, 42% (*n* = 56) only in the gut (rectum/feces), and 33% (*n* = 44) in both the nose and gut of the same infant. Strains that were retrieved exclusively from one site (nose or gut) were usually transient or unclassified, and only 11% of these strains were persistent colonizers. This was in striking contrast to the strains that appeared both in the nose and gut, among which 50% were persistent at both sites (*P* ≤ 0.001, Fisher’s exact test) (Table 2). Thus, there appears to be a strong linkage between being able to colonize two different sites and having the capacity to persist in the microbiota.

**TABLE 2:** Colonization behaviors of the 132 *S. aureus* strains retrieved from the nose and/or gut of infants who were monitored from 3 days to 3 years of age^*a*^

Colonization site	Colonization behavior [*n* (%)] by strain type								
	Persistent			Transient			Unclassified		
	Nose	Gut	Both	Nose	Gut	Both	Nose	Gut	Both
Nose only (*n* = 32)	4 (12.5)			12 (37.5)			16 (50)		
Gut only (*n* = 56)		6 (11)			20 (36)			30 (54)	
Nose and gut (*n* = 44)	28 (64)	33 (75)	22 (50)	6 (14)	2 (4.5)	1 (2)	10 (23)	9 (20)	4 (9)

a Persistent strains appeared in several nasal and/or gut samples over a sampling period of 3 weeks or more. Transient strains colonized the nose and/or gut of an infant for <3 weeks or were present only once at either 1 month or 2 months of age. Unclassified strains were those isolated on a single sampling occasion during a period of long sampling intervals (at 4, 6, 12, 18, or 36 months of age). These strains could not be defined as either persistent or transient.

### Virulence gene pattern in relation to site of colonization.

To determine whether there was any correlation between virulence gene carriage and site of colonization, principal component analysis (PCA) was applied. As shown in Fig. 2c, the *agr3* type, the toxin gene *tst*, and the adhesin gene *bsp* appeared to be enriched in strains that were found only in the nose. Furthermore, strains that were found in both the nose and gut appeared more similar to those found in the gut only, and the *fnbA* gene seemed to be associated with the gut-colonizing strains (Fig. 2c).

This aspect was further explored using orthogonal projection onto latent structures-discriminant analysis (OPLS-DA), which is a regression variety of PCA, whereby virulence gene carriage (*X* variables) was related to the site of bacterial isolation (*Y* variables). This analysis confirmed that the “nose & gut” and “gut-only” strains were similar, as they clustered together at the right side of the plot, and that they were enriched for the *fnbA* and *fnbB* genes (Fig. 2d). Conversely, the “nose-only” strains were characterized as having the *agr3* allele and carrying the adhesin gene *bsp* as well as the genes for the superantigens TSST-1 and SEH.

In the univariate analyses (Fisher’s exact test), the “nose-only” strains (*n* = 32) were compared with strains that colonized the gut, regardless of whether they were also isolated from the nose (*n* = 100). Confirming the multivariable analyses, “nose-only” strains were found to be significantly more likely to carry the superantigen gene *seh* (corrected *P* value [*P*_c_] of 0.043) and tended to more frequently carry the *tst* gene (*P*_c_ = 0.055) and the *agr* 3 allele (*P*_c_ = 0.09) compared to strains obtained from the gut (Table 3). Conversely, the gut-colonizing strains significantly more frequently carried the *fnbA* gene compared to the “nose-only” strains (*P*_c_ = 0.027) (Table 3).

**TABLE 3:** Carriage rates of genes that encode toxins and adhesins in S. aureus strains that colonize only the nose and those that colonize the gut or both the gut and nose^*a*^

Virulence trait	Gene	Carriage rate (% of strains)			
		Only nose (*n* = 32)	Gut (*n* = 100)	*P* value	
				Fisher’s	Corrected
Bone sialoprotein-binding protein	*bsp*	41	19	0.018	0.16
Collagen-binding protein	*cbp*	59	64		
Elastin-binding protein	*ebp*	80	68		
Fibrinogen-binding protein	*fib*	55	58		
Fibronectin-binding protein A	*fnbA*	50	80	0.002	0.027
Fibronectin-binding protein B	*fnbB*	81	93		
Superantigens					
Enterotoxin A	*sea*	6	6		
Enterotoxin C	*sec*	19	29		
Enterotoxin H	*seh*	31	9	0.003	0.043
Enterotoxin K	*selk*	3	8		
Enterotoxin L	*sell*	31	37		
Enterotoxin M	*selm*	56	63		
Enterotoxin O	*selo*	71	71		
Enterotoxin P	*selp*	0	12		
Enterotoxin R	*selr*	9	7		
Toxic shock syndrome toxin TSST-1	*tst*	41	15	0.005	0.055
No superantigen		12	13		
Other toxins					
Beta-hemolysin	*hlb*	44	64		
Epidermal cell differentiation inhibitor toxin	*edin*	3	10		
*agr* alleles					
* agr1*		50	56		
* agr2*		10	20		
* agr3*		40	15	0.008	0.09
* agr4*		0	8		

a Subjects were infants who were monitored from 3 days to 3 years of age. Rates of virulence gene carriage by S. aureus strains that were found exclusively in the nose (*n* = 32) or in the gut (either only in the gut or in both the nose and gut, *n* = 100), isolated from 54 Swedish infants, are shown. Proportions were compared using Fisher’s exact test. The column heading *P* value corrected refers to the *P* values derived after correction for multiple inferences using the Westfall-Young permutation method. No superantigen refers to a lack of carriage of all the screened superantigen genes. The following toxin genes were identified in <5% of the strains: *sed* (enterotoxin D), *eta*, *etb*, and *etd* (exfoliative toxins A, B and D), *pvl* (Panton-Valentine leukocidin), and *lukM* (leukotoxin M). No strain carried the toxin gene *seb* or *selq.* The following adhesin genes were carried by ≥98% of the strains: *clfA* and *clfB* (clumping factors A and B, respectively) and *lbp* (laminin-binding protein).

No significant differences in virulence gene carriage were found between the “gut-only” and “nose & gut” strains in the univariate analysis (data not shown).

A virulence score was calculated for each strain by summing the number of virulence genes carried (among those genes that were screened). The “nose-only” strains did not differ significantly from the strains colonizing the gut (mean, 9.7 versus 10, *P* = 0.2; Mann-Whitney *U* test). Furthermore, the “gut-only” and “nose & gut” strains did not differ from one another in terms of virulence score (10 versus 10, *P* = 0.3).

### Virulence gene patterns and capacities to persist in the nose or gut microbiota.

**FIG 3:**
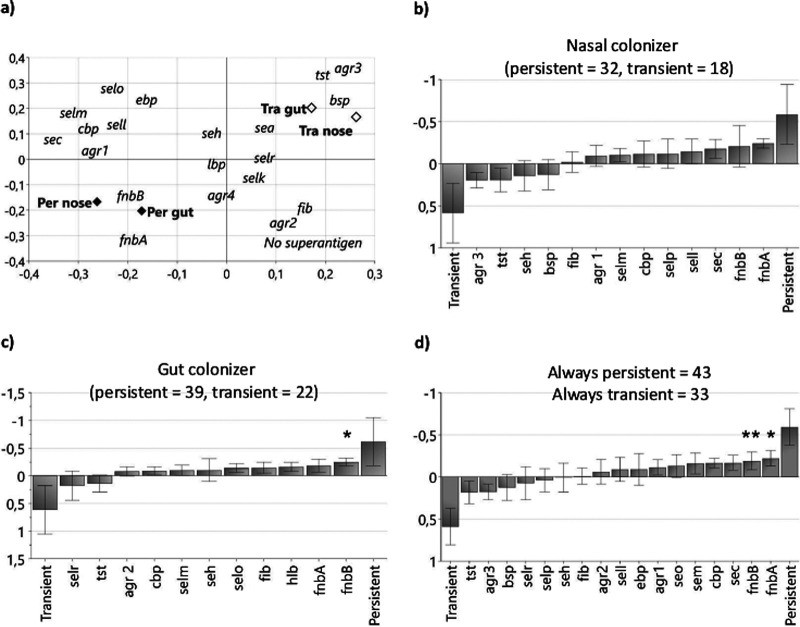
Virulence gene carriage of S. aureus strains in relation to persistence in the infant nose and gut. (a) PCA showing S. aureus virulence gene carriage by nasal- and gut-persistent strains (colonizing the site for ≥3 weeks) and the nasal- and gut-transient strains (colonizing the site for <3 weeks). (b) OPLS-DA analysis showing the links between persistence and virulence gene carriage when including nasal-persistent and nasal-transient S. aureus strains, (c) gut-persistent and gut-transient S. aureus strains, and (d) “always persistent” and “always transient” S. aureus strains. “Always persistent” strains were defined as strains that were persistent at one site and persistent, unclassified or absent at the other site. “Always transient” strains were defined as those that were transient at one site and either transient or absent at the other site. In these OPLS-DA analyses, the characteristics of the strains, i.e., being persistent or transient, are modeled as *y* variables, and the virulence genes associated with persistence appear on the right side of the diagram. Conversely, transient strains and the genes enriched in these strains appear on the left side. Asterisks above a variable bar indicate that this variable is significantly associated with being persistent in a univariate analysis (Fisher’s exact test) after correction for multiple comparisons.

We next examined whether persistence in the nose or gut was associated with any particular virulence traits by comparing persistent and transient strains at these sites (Table 2). PCA indicated that persistent strains resembled one another, regardless of their site of isolation, and that they were enriched for *fnbA* and *fnbB* (Fig. 3a). Conversely, transient strains, regardless of their site of isolation, belonged to the *agr3* allele and carried the *bsp* and *tst* virulence genes (Fig. 3a). It is noteworthy that these same traits were enriched also in the “nose-only” strains (Fig. 2b).

The OPLS-DA analysis revealed that *fnbA* and *fnbB* characterized persistent strains in both the nose (Fig. 3b) and the gut (Fig. 3c). The contribution of *fnbB* to persistence was of similar magnitude at both sites, although it was only significant for gut strains (Fig. 3b and c). This finding might be attributable to the slightly higher numbers of gut strains. Interestingly, nasal-transient strains (left side of diagram in Fig. 3b) tended to belong to the *agr3* allele and to carry the *tst*, *bsp*, and *seh* genes, i.e., the same traits that characterized the “nose-only” strains (Fig. 2d).

We speculated that the poor capacity of *tst-agr3-bsp-seh* strains to persist could be due to a lack of genes that promoted persistence, e.g., *fnbAB*. Therefore, we analyzed the association between *fnbA* or *fnbB* on the one hand and *tst*, *bsp*, *seh*, and the *agr3* allelic variety on the other hand (*n* = 132 strains). There was, indeed, a strong inverse relationship between *tst* and *fnbA* carriage, i.e., 21% of the *tst*-positive strains (6/28) carried *fnbA*, while this also was true in the case 86% of *tst*-negative strains (90/104, *P* = 0.0001) and between *tst* and *fnbB* carriage (71% versus 95%, *P* = 0.001). The same correlations were seen for *agr3* and *fnbA* (26% versus 87%, *P* = 0.0001), *agr3* and *fnbB* (78% versus 95%, *P* = 0.01), and *bsp* and *fnbA* (41% versus 83%, *P* = 0.0001) but not for *bsp* and *fnbB* (84% versus 92%, *P* = 0.3). There were no significant associations between *fnbA* or *fnbB* carriage and *seh* carriage.

As the capacity to persist for both gut-colonizing and nose-colonizing strains seemed to be linked to the same traits, we compared strains that were always persistent colonizers to those that always displayed a transient colonization pattern, regardless of their colonization site. We identified 43 strains that were “always persistent,” i.e., they were persistent at one site or both and not transient at any site (they could be unclassified or absent at the other site). Of these, 22 were persistent both in the nose and gut, and 9 were present only in the nose and 12 only in the gut (Table S1). We compared these with 33 “always transient” strains (defined as strains that were transient at one site and transient or absent at the other site), and 12 of these strains were found (transiently) only in the nose, 20 only in the gut, and 1 transient in both the nose and gut (Table 2). As shown in Fig. 3d, the *fnbA* and/or *fnbB* genes were enriched in the strains that were “always persistent,” and this was confirmed in the univariate analysis (Table 4). In fact, all of the strains that were classified as “always persistent” carried the *fnbB* gene (Table 4). The association between a strain always being transient and its carriage of *agr3* and *tst* was found to be not significant in the univariate analysis (Table 4).

**TABLE 4:** Prevalence rates of genes that encode various toxins and adhesins in persistent and transient strains that colonized the nose and/or gut^*a*^

Virulence trait	Gene	Strains		*P* value	
		Always persistent^*b*^ (*n* = 43)	Always transient^*c*^ (*n* = 33)	Fisher’s	Corrected
Adhesins					
Bone sialoprotein-binding protein	*bsp*	26	36		
Collagen-binding protein	*cbp*	72	51		
Elastin-binding protein	*ebp*	79	66		
Fibrinogen-binding protein	*fib*	57	37		
Fibronectin-binding protein A	*fnbA*	84	48	0.001	0.01
Fibronectin-binding protein B	*fnbB*	100	73	0.0002	0.002
Superantigens					
Enterotoxin A	*sea*	5	0		
Enterotoxin C	*sec*	37	18		
Enterotoxin H	*seh*	12	12		
Enterotoxin L	*sell*	48	33		
Enterotoxin M	*selm*	72	45	0.03	0.22
Enterotoxin O	*selo*	77	66		
Enterotoxin P	*selp*	9	6		
Enterotoxin R	*selr*	5	12		
Toxic shock syndrome toxin TSST-1	*tst*	16	36		
No superantigens		7	21		
Others					
Beta-hemolysin	*hlb*	62	48		
Epidermal cell differentiation inhibitor toxin	*edin*	9	3		
*agr* alleles					
* agr1*		57	53		
* agr2*		19	9		
* agr3*		17	37		
* agr4*		7	0		

a Subjects were infants who were monitored from 3 days to 3 years of age. Proportions were compared using Fisher’s exact test. The term *P* value corrected refers to the *P* values derived after correction for multiple inferences using the Westfall-Young permutation method. No superantigen refers to a lack of all the screened superantigen genes. Data regarding the toxin/adhesin genes that were identified in <0.5% or in ≥98% of the strains are explained in a footnote to Table 2.

b Rates of virulence gene carriage for the 43 strains that were persistent at one site and either persistent, unclassified, or absent at the other site.

c Rates of virulence gene carriage for the 33 strains that were transient at one site and either transient or absent at the other site.

We also compared the virulence scores for the “always persistent” and “always transient” strains. The “always persistent” strains had significantly higher virulence scores than the “always transient” strains (mean, 11 versus 9; *P* = 0.002).

### Duration of colonization of persistent S. aureus strains in the nose and gut.

We compared the duration of colonization of those strains that were persistent colonizers of the nose or gut. Overall, persistent colonization was noted for 32 strains in the nose and 39 strains in the gut (Table 2). The average duration of colonization of the persistent nasal strains was ≥26 weeks, compared to ≥42 weeks for persistent strains in the gut (*P* = 0.001).

Furthermore, among the strains that persisted at both sites (*n* = 22; Table 2), the duration of persistence was significantly longer in the gut (mean, ≥49 weeks) than in the nose (mean, ≥20 weeks) (*P* = 0.003).

### Fecal population counts of gut-colonizing S. aureus strains in relation to gut persistence and virulence gene carriage.

The S. aureus counts at different fecal sampling occasions were compared between persistent and transient gut-colonizing strains, and no significant differences were observed. Carriage of *fnbA* or *fnbB* was not associated with increased fecal counts at any of these time points. Since carriage of genes that encode collagen-binding protein, enterotoxin M, or enterotoxin O was previously linked to high fecal counts of S. aureus (23), we also analyzed these genes. However, no significant associations were observed. Furthermore, no correlation was found between the virulence score of a strain and its fecal population counts (data not shown).

### S. aureus strain characteristics in relation to the lifestyle factors and clinical histories of the colonized infants.

The following lifestyle factors and clinical histories were analyzed in relation to S. aureus strain virulence and colonization characteristics: gender, delivery mode, intrapartum antibiotic exposure, antibiotic treatment (at least once between birth and 18 months of age), exclusive breastfeeding for at least 4 months, having elder siblings, being from farming families, and pet exposure (Table 1). Carriage of *fnbA* or *fnbB* or the virulence score of the S. aureus strain did not correlate significantly with any of these factors. Furthermore, none of these factors significantly affected the proportions of persistent versus transient strains in the nose and gut of colonized infants. Regarding the long-term persistence of S. aureus strains, the duration of colonization for persistent nasal strains was significantly shorter in infants with elder siblings than in infants without siblings (mean, 7 versus 30 weeks, *P* = 0.01); the same was true for persistent gut strains (mean, 26 versus 51 weeks, *P* = 0.02). However, the statistical significance disappeared following multiple inference correction.

## DISCUSSION

In this study, we monitored nasal and gut colonization by S. aureus in parallel in a birth cohort of 65 infants who were monitored from 3 days to 3 years of age.

In total, 83% of the infants yielded at least one positive sample, and 71% had S. aureus at both sites. Although nasal and gut colonization increased in parallel during the first month of life, nasal colonization declined thereafter, and between 6 and 18 months of age, S. aureus was isolated 2 to 4 times more frequently from the feces than from the anterior nares.

Nasal samples were obtained using swabs. This method was also used for assessing rectal colonization on day 3 after delivery. Thereafter, gut colonization was assessed by quantitative culturing of feces. The latter method could be considered more sensitive, although in the case of enteric pathogens the culturing of rectal swabs is as sensitive as the culturing of feces (32). There is no reason to believe that this is not also true for S. aureus. Furthermore, there was no difference in the nasal and gut colonization rates up to 1 month of age, and thereafter nasal colonization decreased while gut colonization remained high. A decline in nasal colonization by S. aureus after the first weeks of life has been noted previously (33). The observed decrease may relate to anatomical changes, increased production of antimicrobial peptides, and increased competition from other skin colonizers. Gut colonization by S. aureus decreased at a much later stage, after 1 year of age, and this probably reflects increased competition from the more complex gut microbiota (34).

Other groups have suggested that the gut is the main habitat of S. aureus strains under certain circumstances (35). Piewngam and Otto have even suggested that gut colonization by S. aureus is the primary event and nasal colonization is secondary and transient, resulting from inoculation of the nose with gut-colonizing strains, representing anorectal-nasopharyngeal transmission (35). Such transmission might explain the failure of topical decolonization when only the nose is targeted (7, 36).

Our previous and present results confirm that successful isolation of S. aureus from the feces is not simply the result of the gut being seeded by the nasal microbiota. First, the high population counts of S. aureus during early infancy, 10^7^ CFU/g feces on average (9), can hardly be achieved without the multiplication of bacteria indigenous to the gut. Second, in the present study, we identified almost twice as many strains that were found only in the feces (*n* = 56) than strains that were found only in the nose (*n* = 32). Third, strains that colonized, for at least 3 weeks, both the gut and the nose of the same infant persisted, on average, more than twice as long in the gut as in the nose (≥49 weeks compared to ≥20 weeks, on average). Fourth, the strains that colonized both the gut and the nose of the same individual displayed fecal population counts that were no higher than those of the strains that colonized only the gut of an infant (our unpublished observations). We conclude that S. aureus is a true gut colonizer of young infants and that, at least in infants and young children, gut colonization by S. aureus is as common as or more common than nasal colonization.

Persistent carriage in both the nose and gut of the same S. aureus strain was frequently observed in our cohort. Interestingly, 50% of the strains that were retrieved from both the nose and gut of an infant were persistent at both sites, while only 13% of the strains found exclusively in the nose and 11% of the strains found exclusively in the gut colonized that site for ≥3 weeks (*P* = 0.001). Thus, for S. aureus, there is a strong correlation between being able to colonize two different ecological niches (nose and gut) and being able to persist for extended periods of time in the microbiota.

Fibronectin-binding proteins A and B appeared to be associated with long-term persistence within either the nasal or gut microbiota. In fact, when we defined a subset of strains that were always persistent, whether they colonized the nose or the gut or both, and never appeared transiently at any of these sites, 100% of them carried the *fnbB* gene. Moreover, *fnbB* gene carriage was also significantly associated with persistence among the gut-colonizing strains. We regard the higher prevalence rates of the *fnbA* and *fnbB* genes among persistent strains as indicating that S. aureus exploits these proteins during long-term colonization of the nose and/or gut of infants. Accordingly, preferential transcription of the *fnbA* gene was observed in S. aureus strains in the noses of healthy carriers (22). As fibronectin is an extracellular matrix protein, it is not thought to be present on intact epithelia. However, the fibronectin-binding adhesin may recognize other (as-yet unknown) receptors, given that S. aureus binds to intact mammary gland epithelial cells via fibronectin-binding protein in a manner that can be inhibited by soluble fibronectin (37). To the best of our knowledge, the importance of fibronectin-binding protein for persistent colonization of S. aureus in the nose and gut has not previously been demonstrated. Animal studies have shown that fibronectin-binding proteins play essential roles in the capacities of S. aureus to cause septicemia, septic arthritis, and endocarditis (15, 17, 18).

In the present study, carriage of the *fnbA* gene was also significantly linked to the ability of S. aureus strains to colonize the gut. Strains that were restricted to the nose were instead enriched for the *tst*, *bsp*, and *seh* genes and often belonged to the *agr3* allelic genotype. These traits were, however, not associated with persistent colonization of the nose but rather with a transient colonization behavior. Strains that produce TSST-1 are mostly of the *agr3* allelic variety and originate from a single clone (38). This clone may be a poor colonizer of infants, perhaps being particularly inept at surviving in the gut milieu. This could relate to lacking the *fnbA* gene, as there was a significant inverse relationship between the carriage of *tst* and the carriage of *fnbA*: only about 20% of the *tst*-positive strains carried the *fnbA* gene, while 86% of the *tst*-negative strains carried the *fnbA* gene. TSST-1 itself is unlikely to function in the gut, as it is susceptible to cleavage by pepsin and, therefore, is likely to be structurally unstable in this environment (39, 40).

Nasal colonization by S. aureus has previously been linked to the presence of ClfB (25, 26), which promotes staphylococcal adherence to human type I cytokeratin (25). In the present study, 99% of all the nasal and intestinal S. aureus strains carried the genes for clumping factors A and B, making it impossible to assess the impact of ClfB on persistent colonization by S. aureus strains.

The superantigens SElM, SElO, SEG, and SEI, which are all encoded by the *egc* locus, have been suggested to play a role in S. aureus nasal colonization (20, 31). We have previously observed that strains carrying the genes for SElM or SElO attained higher counts in the feces of colonized infants than strains that are negative for these genes (23). In the present study, carriage of *selm* was more common in strains that were “always persistent” at the site that they colonized than in strains that were “always transient” at the site where they appeared. However, the statistical significance of this difference was lost after correction for multiple comparisons.

Interestingly, we found that the “virulence score” of a strain, i.e., the number of different virulence genes identified in the strain, was significantly positively related to the capacity of that strain to persist at the site that it colonized, either in the gut or the nose. This supports the notions that virulence factors have evolved primarily to facilitate commensal colonization and that successful commensal colonization and pathogenicity are linked.

Although nasal colonization increases the risk for invasive infection 3-fold (6), gut carriage is also an important risk factor for S. aureus infections (7, 11, 12). Interestingly, patients who are colonized by S. aureus in both the nose and gut are more likely to contract S. aureus infection than patients who are colonized only in the nose (12). This accords with our observations of a strong link between the capacity of a strain to colonize both sites and its ability to establish long-term persistence, which, in turn, is linked to a high “virulence score,” i.e., the possession of not only *fnbA* and *fnbB* but also other virulence traits.

Our examined cohort is relatively small and not designed to elucidate potential connections between S. aureus carriage and infection. Although we do not have access to hospital records, two infants (out of 65) received treatment with isoxazolyl penicillin (one of them twice) during the first 18 months of life, indicating S. aureus infection. One of these infants, who received treatment with this antibiotic on one occasion between the ages of 12 and 18 months, carried one nasal-persistent and one gut-persistent S. aureus strain that colonized the nose/gut from 1 month of age up to 3 years of age. The other infant was not colonized with S. aureus.

S. aureus colonization may be affected by different demographic, clinical, and behavioral parameters. Indeed, we found a negative association between being breastfed and yielding at least one sample (nasal or rectal/fecal) that was positive for S. aureus. This might be explained by the array of antibacterial factors, such as lysozyme and lactoferrin, present in breast milk. In a separate study based on the same birth cohort, we have uncovered a relationship between different lifestyle parameters and colonization by various gut bacteria, including S. aureus (A. Ljung, B. Hesselmar, H. Rabe, F. L. Nowrouzian, S. Johansen, A. E. Wold, and I. Adlerberth, unpublished data). For example, being the first-born child was found to be associated with an increased likelihood of S. aureus gut colonization. In the present study, we have focused on the colonization behaviors of S. aureus strains and noted that persistent nasal and gut strains resided at these sites for a longer period in first-born infants than in infants with elder siblings. These two observations are in agreement and suggest that S. aureus has greater difficulties colonizing and persisting in infants who have elder siblings. This indicates a more rapid maturation of the commensal microbiota in infants with elder siblings, leading to an ecological pressure on, for example, S. aureus, which may reduce the ability of strains to persist at mucosal sites.

We have previously observed that certain adhesins and other traits that are commonly regarded as virulence factors in uropathogenic Escherichia coli also promote the commensal persistence of E. coli strains in the infantile gut (41–43). S. aureus fibronectin-binding proteins, and probably other virulence factors, may well exert such a dual function, initially enhancing commensal nasal and gut colonization and thereafter aggravating disease when there is invasion.

## MATERIALS AND METHODS

### Subjects.

The FARMFLORA birth cohort comprises 65 infants from rural areas of southwest Sweden. The cohort was recruited and monitored to identify factors including early microbiota colonization associated with farm living and contributing to protection against allergy development; 28 of the infants were from farming families, and 37 were from nonfarming control families (44). The study was approved by the Ethics Committee of the University of Gothenburg (Dnr. 363-05). The characteristics of the cohort are listed in Table 1.

### Sampling and isolation of S. aureus from infant nasal and gut microbiota.

Briefly, the nasal microbiota was sampled using a cotton-tipped swab (Copan Diagnostics Inc., CA, USA) at the following time points: 3 days; 1, 2, 4, and 8 weeks; and 4, 6, 12, 18 and 36 months of age. On the same occasions, the gut microbiota was sampled, either through the use of a rectal swab (at 3 days of age) or through the collection of fecal samples (all other occasions). The methodology used for the culturing and identification of S. aureus has been described previously (45). In brief, swabs were streaked onto Staphylococcus agar plates, while a calibrated spoonful of feces was serially diluted in sterile peptone water in 10-fold steps and the dilutions were plated onto Staphylococcus agar (10). After incubation aerobically at 37°C for 48 h, colonies that differed with respect to size, shape, or color were separately enumerated from plates and identified as Staphylococcus based on Gram staining and catalase production. S. aureus was identified by a positive coagulase test. The lowest level of detection was 330 CFU/g feces. The rectal swab cultures were not quantified. The S. aureus fecal population counts from 1 week to 18 months of age are reported elsewhere (Ljung et al., unpublished). Here, the fecal population counts for each individual S. aureus strain in each stool sample were determined.

### Strain typing of S. aureus.

Overall, 83% (54/65) of the infants yielded at least one positive S. aureus culture. In total, 449 S. aureus isolates were obtained from these infants: 186 from nasal swabs and 263 from rectal swabs/fecal samples. Random amplified polymorphic DNA (RAPD) analysis was used to identify different strains in the same individual, as described previously (9, 23). All the S. aureus nasal and gut isolates from an individual infant were assessed together in a single run or two consecutive runs. Isolates that yielded identical RAPD patterns were regarded as belonging to a single strain, regardless of the site of isolation, and they were labeled with the infant number and a letter (strains 1A, 1B, and 1C in infant 1, strains 2A to D in infant 2, and so forth; the RAPD patterns were not compared between infants).

Strains were designated “persistent” at one site if they appeared in several samples from that site (nose or gut) over a period of 3 weeks or longer. The length of the persistence period was calculated as the amount of time that elapsed between the first and last sample from which the strain was retrieved. “Transient” strains were defined as strains that were present at a site for <3 weeks, while strains that were retrieved only once at either 1 month or 2 months of age were also defined as transient. “Unclassified strains” were those that appeared in a single sample obtained at 4, 6, 12, 18, or 36 months of age, as they could not be unambiguously categorized as either persistent or transient due to the long interval between the sampling occasions. This classification of strains has been used in previous studies (9, 23, 46).

### S. aureus virulence genes.

A representative isolate of each strain was analyzed using multiplex PCRs (23) for the accessory gene regulatory (*agr1* to *-4*) locus and genes encoding the following adhesins: fibrinogen-binding protein, ClfA and ClfB, elastin binding-protein, laminin-binding protein, collagen-binding protein, bone sialoprotein-binding protein, and fibronectin-binding proteins A and B. Furthermore, genes encoding the following S. aureus toxins were identified: enterotoxins SEA to SED and SEH, SElK, SElL, SElM, SElO, SElR, SElP, SElQ, TSST-1, exfoliative toxins A, B, and D, β-hemolysin, epidermal cell differentiation inhibitor toxins A to C, Panton-Valentine leucocidin, and leukotoxin M. A virulence score was calculated for each strain by summing the number of virulence genes carried.

The virulence gene profiles of the S. aureus strains that colonized the present cohort between 3 days and 2 months of life have previously been reported and analyzed in relation to the development of eczema in colonized infants (45).

### Statistical analysis.

Proportions were compared using Fisher’s exact test. The Westfall-Young permutation method was used to correct for multiple inferences (47).

The Mann-Whitney *U* test was used to compare the S. aureus fecal population counts or the virulence scores between different groups of strains. This test also was used to compare the time of colonization (in weeks) of persistent strains at different body sites. Spearman’s correlation was used to test for correlation between the S. aureus fecal population counts on different sampling occasions and virulence scores (SPSS software; IBM Corp., NY, USA).

Principal component analysis (PCA) and orthogonal projection onto latent structures-discriminant analysis (OPLS-DA), which is a regression development of PCA, were used to obtain an overall picture of the virulence gene carriage pattern and its relationships to the site of isolation and persistent or transient colonization in the microbiota (SIMCA-P; Umetrics AB, Umeå, Sweden).
